# Optimization of Fermentation Conditions and Bench-Scale for Improvement of a Novel Glycoprotein GP-1 Production by *Streptomyces kanasenisi* ZX01

**DOI:** 10.3390/molecules23010137

**Published:** 2018-01-10

**Authors:** Yong Zhou, Xin Zhou, Dai-Lin Yu, Bu Sang, Jun-Tao Feng, Li-Rong Han, Xing Zhang

**Affiliations:** 1 Research and Development Center of Biorational Pesticide, Northwest Agriculture & Forestry University, Yangling 712100, China; zy890619@gmail.com (Y.Z.); xinchou@nwafu.edu.cn (X.Z.); fengjt@nwsuaf.edu.cn (J.-T.F.); 2 Agriculture Research Institute, Tibet Academy of Agricultural and Animal Husbandry Science, Lhasa 850032, China; yudailinpz@126.com (D.-L.Y.); sangbu007@foxmail.com (B.S.); 3 Shaanxi Research Center of Biopesticide Engineering & Technology, Yangling 712100, China

**Keywords:** response surface methodology, glycoprotein, *Streptomyces*, anti-TMV, optimization

## Abstract

GP-1 is a novel glycoprotein produced by *Streptomyces kanasenisi* ZX01 that was isolated from soil near Kanas Lake with significant bioactivity against tobacco mosaic virus. However, extremely low fermentation production has largely hindered further research and market applications on glycoprotein GP-1. In this study, response surface methodology was used to optimize fermentation conditions in a shake flask for higher glycoprotein GP-1 production. When the optimized fermentation conditions were inoculum volume of 6%, initial pH of 6.5, and rotating speed of 150 rpm, glycoprotein GP-1 production could reach 0.9253 mg/L, which was increased by 52.14% compared to the original conditions. In addition, scale-up fermentation was conducted in a 5-L bioreactor to preliminarily explore the feasibility for mass production of glycoprotein GP-1 in a large fermentor, obtaining GP-1 production of 2.54 mg/L under the same conditions, which was 2.75 times higher than the production obtained from a shake flask of 0.9253 mg/L. This work will be helpful to improve GP-1 production on a large scale and lay the foundations for developing it as a novel agent against plant virus.

## 1. Introduction

As an important part of natural products, microbial metabolites exhibit high diversity in producing species, functions, bioactivities and chemical structures, which have gradually become a hot spot of scientific research in recent years [1]. *Streptomyces* is a common and famous species of microorganism, and metabolites isolated from *Streptomyces* account for nearly half of the total number of microbial compounds [1,2,3]. Antibiotics and similar small molecular compounds are an important group of metabolites produced by *Streptomyces* and usually show various biological activities, representing the main topic of research and literature [4,5]. With the rapid progress of methods and technologies of screening and isolation, the number of novel microbial metabolites continues to grow but a number of biological macromolecules, such as polysaccharide, polypeptide and glycoprotein, were also discovered successively [6,7,8,9,10]. The biological activities of these bio-macromolecules are more diversified due to their chemical structural complexity.

*Streptomyces kanasenisi* ZX01 (CGMCC 4893) was isolated from soil around Kanas Lake, Xinjiang Province, China. A novel glycoprotein GP-1 was isolated from fermentation broth of strain ZX01 with significant bioactivity against some plant viruses, especially tobacco mosaic virus (TMV) [11]. Glycoprotein GP-1, with the molecular weight of approximately 8.5 kDa, contains 40.23% carbohydrates and 54.36% polypeptides with N-linked and O-linked glycan [12,13]. Our previous study indicated that glycoprotein GP-1 production was strongly affected by medium components, especially the types and concentrations of carbon and nitrogen sources [14]. Process factors (temperature, pH, rotating speed) also play an important role in improving objective metabolites production and may contribute to the reduction of fermentation cost, even changing the path of microbial biosynthesis to discover new natural products [15,16,17].

Various statistical design methods can be used to optimize the fermentation process [18]. The conventional method of one-factor-at-a-time optimization can only change one independent variable with other variables kept constant, which is laborious and time-consuming. Furthermore, this method is not guaranteed to obtain the optimal values of variables and is unable to describe the interactive effects among different variables [18,19]. The disadvantages of the one-factor-at-a-time method can be overcome by response surface methodology (RSM). Nowadays, optimization through RSM has been commonly applied to various fields of science, such as agriculture, engineering and food [20,21,22,23]. RSM is a collection of mathematical and statistical techniques based on the fit of a polynomial equation to the experimental data, which can determine the optimal levels of the variables for a desirable response, and estimate the interactions between a set of controlled experimental variables [18].

The objective of this work was to optimize the fermentation conditions (inoculum volume, initial pH and rotating speed) of *Streptomyces kanasenisi* ZX01 in a shake flask for the maximum production of glycoprotein GP-1 using response surface methodology. In addition, scale-up fermentation was carried out in 5 L bioreactor to preliminarily explore the feasibility for mass production of glycoprotein GP-1 in a large fermentor.

## 2. Results

### 2.1. Effects of Inoculum Volume, Initial pH and Rotating Speed on Glycoprotein GP-1 Production

**Figure 1:**
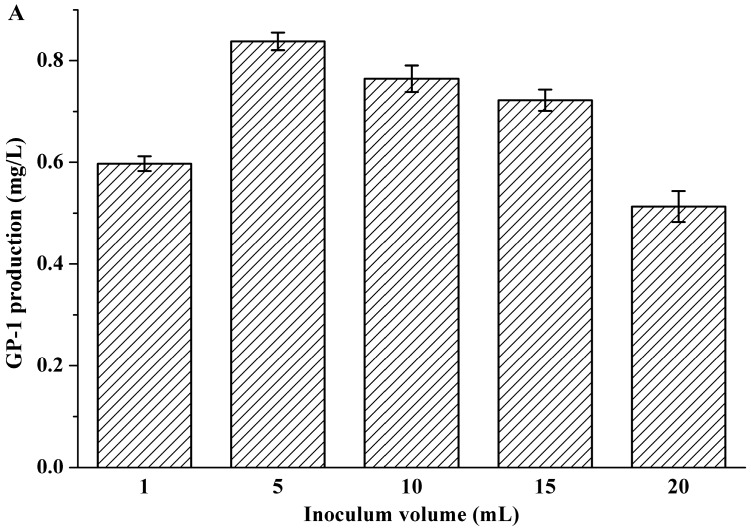

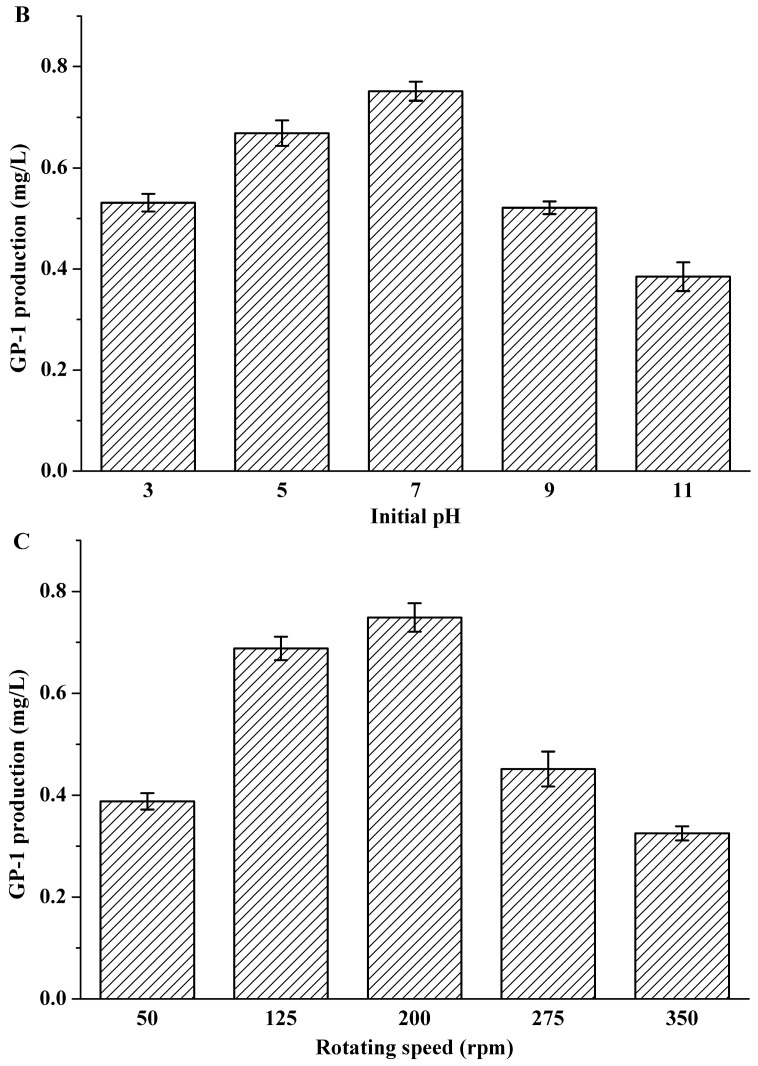
Effects of inoculum volume (**A**); initial pH (**B**) and rotating speed (**C**) on glycoprotein GP-1 production.

The equation fitted by response surface methodology can accurately predict the highest response value while being established in the area near the central point of variables [24]. Therefore, a preliminary experiment was performed in order to confirm the central values of the variables in central composite design when other conditions were controlled at inoculum volume of 10%, initial pH of 7.0 and rotating speed of 200 rpm (Figure 1). The results show that the central values of three variables were inoculum volume of 5%, initial pH of 7.0, and rotating speed of 200 rpm, respectively.

### 2.2. Optimization of Fermentation Conditions by RSM

After confirming the central points of inoculum volume, initial pH and rotating speed, Table 1 presents the levels of three variables designed by response surface methodology based on central composite design (CCD). A total of 20 experimental combinations were performed (Table 2). The experimental values of GP-1 production were results of three replicates, while the predicted values were obtained from the following quadratic polynomial analyzed by Design Expert 8.0:

(1) $$Y=0.83+0.025x_{1}-0.069x_{2}-0.011x_{3}+0.013x_{1}x_{2}-0.00895x_{1}x_{3}-0.017x_{2}x_{3}-0.030x_{1}^{2}-0.053x_{2}^{2}-0.053x_{3}^{2}$$

where *Y* is the predicted value of GP-1 production, *x*_1_, *x*_2_ and *x*_3_ are coded values of inoculum volume, initial pH and rotating speed, respectively. Coded values and actual values can be switched by the following equation:

(2) $$x_{1}=\frac{\left(X_{1}-5\right)}{2};\;x_{2}=\frac{\left(X_{2}-7\right)}{1};\;x_{3}=\frac{\left(X_{3}-200\right)}{50}$$

In order to evaluate the significance and accuracy of the model, an analysis of variance (ANOVA) was calculated (Table 3). The *F* value and *P* value are main indicators of the significance of model and variables. The larger the *F* value or the smaller the *P* value is, the better the significance and accuracy of the model is [25]. The ANOVA of Table 3 shows that the model is highly significant, due to *F*_model_ = 29.61 > *F*_0.01 (9, 10)_ = 4.94 and *P*_model_ < 00001. On the other hand, the model’s goodness of fit can be checked by the determination coefficient (*R*^2^) and the correlation coefficient (*R*). The *R*^2^ value is always between 0 and 1. The closer the *R*^2^ value is to 1, the better the correlation between the experimental and predicted values is [26]. In this study, the adj-*R*^2^ (0.9638) demonstrates that 96.38% variation in the model is caused by three variables and only 3.62% variation could not be explained by the model.

The model also indicates that the linear terms of $x_{2}$, $x_{3}$ and the quadratic terms of $x_{2}^{2}$, $x_{3}^{2}$ are more significant than others, owing to larger *F* values and smaller *P* values. In other words, the variation of initial pH and rotating speed can affect glycoprotein GP-1 production more greatly and directly.

**Figure 2:**
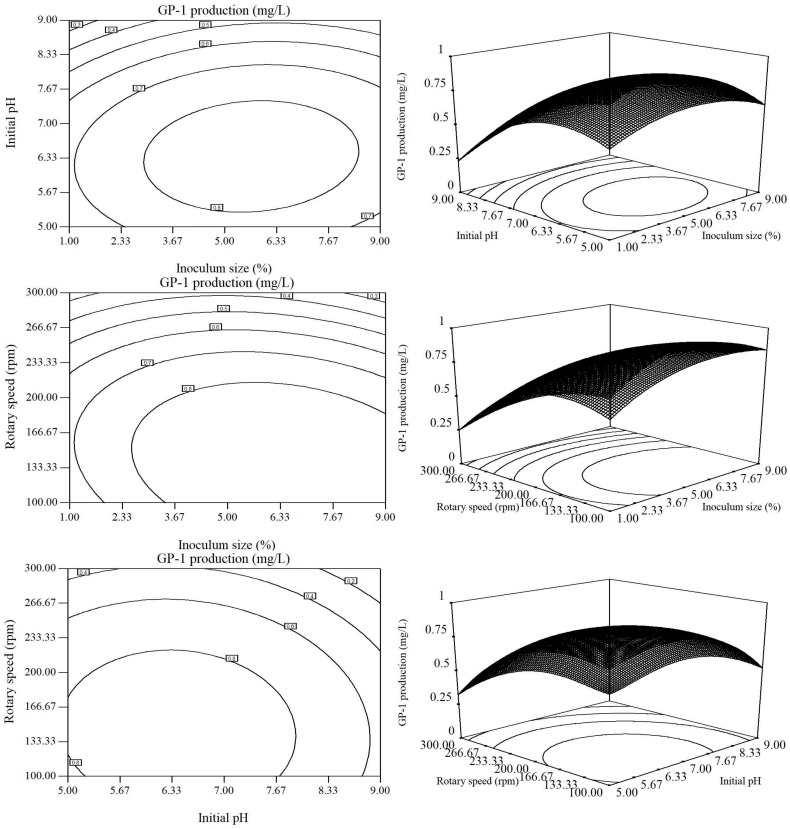
Counter plots and response surface plots of effects of inoculum volume, initial pH and rotating speed on glycoprotein GP-1 production.

By analyzing the model, a series of 2D contour plots and 3D response surface plots were drawn to visually expose the optimal values of variables and the highest response value (Figure 2). Each ellipse in 2D contour plots represents one response value affected interactively by two variables with the third maintained at its zero level. The optimal values of variables could be obtained in the smallest ellipses. From corresponding 3D response surface plots, the maximum response value could be predicted, and the interaction between each variable pair could be understood [27].

As shown in Figure 2, the maximum glycoprotein GP-1 was obtained while the optimal values of inoculum volume, initial pH and rotating speed were in range of 5.00~6.33%, 6.33~7.00 and 133.33~166.67 rpm, respectively. Through analysis of Design Expert software, the optimal values of variables were inoculum volume of 5.94%, initial pH of 6.57 and rotating speed of 147.17 rpm, leading to the maximum glycoprotein GP-1 of 0.9102 mg/L.

### 2.3. Verification of the Model

In order to determine whether the results predicted by the model are consistent with the actual results, a verification experiment with optimized fermentation conditions (inoculum volume 6%, initial pH 6.5 and rotating speed 150 rpm) was performed in triplicate. The actual glycoprotein GP-1 production was 0.9253 mg/L, which is similar to the predicted value of 0.9102 mg/L. This indicates that the model built by RSM could efficiently and accurately optimize fermentation conditions to achieve high glycoprotein GP-1 production by *Streptomyces kanasenisi* ZX01.

In addition, a control experiment was carried out with the original fermentation conditions (inoculum volume 1%, initial pH 7.0 and rotating speed 200 rpm), obtaining the glycoprotein GP-1 production of 0.6082 mg/L and anti-TMV inhibition rate of 68.82%. After optimization, glycoprotein GP-1 production and anti-TMV inhibition rates were increased by 52.14% (0.9253 mg/L) and 24.11% (85.41%), respectively.

### 2.4. Fermentation in a 5-L Bioreactor

**Figure 3:**
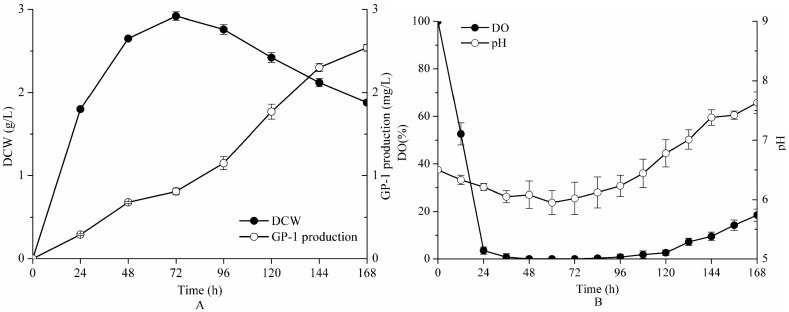
Time courses of *Streptomyces kanasenisi* ZX01 fermentation in a 5-L bioreactor under the optimized fermentation conditions: GP-1 production and dry cell weight (DCW) (**A**), dissolved oxygen (DO) and pH (**B**).

Based on the fermentation conditions (inoculum volume 6%, initial pH 6.5 and agitation speed 150 rpm) obtained from a shake flask, scale-up fermentation was conducted in a 5-L bioreactor. Time courses of GP-1 production, dry cell weight (DCW), dissolved oxygen (DO) and pH are presented in Figure 3.

As shown in Figure 3A, the fermentation process of *Streptomyces kanasenisi* ZX01 was apparently divided into three phases. In the logarithmic phase (0~48 h), glycoprotein GP-1 was synthesized with the rapid growth of cells. In the stationary phase (48~72 h), cell growth and metabolism became relatively stable. In the decline phase (72~168 h), glycoprotein GP-1 was largely secreted from cells into fermentation broth thanks to gradual cell death. DCW reached the maximum value of 3.02 g/L at 72 h, while final GP-1 production was 2.54 mg/L.

Figure 3B presents the changes of DO and pH during the fermentation process. DO concentration was decreased greatly within 24 h due to the consumption of cell growth. During 24~120 h, the DO value always remained at a very low level, as strain utilized oxygen to keep growth and biosynthesize glycoprotein GP-1 and other metabolites at the same time. After 120 h, cells began to die and oxygen demand was reduced, resulting in slow recovery of DO. On the other hand, pH decreased tardily from 6.5 to 6.0 during 0~72 h, and then increased to approximately 7.5.

The results demonstrate that *Streptomyces kanasenisi* ZX01 requires plenty of oxygen during its process of growth and metabolism, which belongs to aerobic microorganism. A shake flask does not provide stable agitation and an aeration system as with a fermentor, resulting in an extremely low level of oxygen supply and transfer. As a result, glycoprotein GP-1 production of 2.54 mg/L on bench-scale was 2.75 times higher than that from a shake flask (0.9253 mg/L), which indicates the feasibility for mass production of glycoprotein GP-1 in a fermentor.

## 3. Discussion

Glycoprotein GP-1 isolated from *Streptomyces kanasenisi* ZX01 was identified to be a novel natural compound with significant bioactivity against TMV [12]. GP-1 can not only destroy TMV particles directly but also induce the system resistance. This has tremendous potential to become a novel anti-TMV agent with low toxicity, a broad spectrum, and high environmental compatibility [13]. However, extremely low fermentation production has largely hindered further research and market applications of strain ZX01, glycoprotein GP-1 and other metabolites [14]. For the above reason, the purpose of this work was to improve glycoprotein GP-1 production through optimizing the fermentation conditions (inoculum volume, initial pH and rotating speed) using response surface methodology.

pH, a very important parameter in the fermentation process, is a comprehensive response to microbial growth and metabolism. The effects of pH on microbial growth and metabolites synthesis are mainly reflected in the following aspects: (1) pH affects enzyme activity. When certain enzyme activity is inhibited in a cell, microbial metabolism will be blocked; (2) pH affects the charge of microbial cell membranes associated with permeability, thus affecting the absorption of nutrients and the excretion of metabolites; (3) pH affects the dissociation of medium components, which further influences strain to absorb and utilize these components; (4) pH can affect the direction of microbial metabolism [28,29,30].

Inoculation also has a great impact on the growth of microorganisms and the accumulation of metabolites. On one hand, excessive inoculation will speed up the consumption of the medium and lead to the strain being reached in advance in the stationary phase and decline phase. On the other hand, low inoculum volume and cell concentration will extend the logarithmic phase [31,32].

For both aerobic fermentation and anaerobic fermentation, the study of dissolved oxygen is significant to enhance productivity and improve product quality. In flask fermentation, the level of dissolved oxygen is generally improved by increasing the rotating speed of the shaker, due to the lack of air supply equipment as with a large fermentor [33].

The optimal values of inoculation volume, initial pH and rotating speed were determined by response surface methodology based on central composite design that was proved to be a powerful and efficient tool to acquire the highest glycoprotein GP-1 production by *kanasenisi* ZX01. After optimization, the final fermentation conditions were inoculum volume of 5.94%, initial pH of 6.57 and rotating speed of 147.17 rpm, respectively. Theoretically, the predicted value of GP-1 production could reach 0.9102 mg/L under these conditions. In practice, GP-1 production was found to be 0.9253 mg/L in the verification experiment with inoculum volume of 6%, initial pH of 6.5 and rotating speed of 150 rpm, which also indicated that the model simulated by RSM was able to predict GP-1 production accurately. Compared to 0.6082 mg/L under the original conditions, glycoprotein GP-1 production was increased by 52.14% after optimization by RSM.

Moreover, a scale-up fermentation of glycoprotein GP-1 was performed in a 5-L fermentor, obtaining GP-1 production of 2.54 mg/L and 2.75-fold increase compared with that in a shake flask. This result indicated that strain ZX01 belongs to aerobe and requires a lot of oxygen for growth and metabolism. The tremendous differences between a shake flask and fermentor can have a great impact on the fermentation process. Unlike a fermentor with stable agitation and an aeration system, oxygen supply in shake flask fermentation mainly depends on the surrounding environment. Although dissolved oxygen in a flask can be improved by adjusting the rotating speed and working volume, the level of DO is still much lower than that in a fermentor. Therefore, DO is the key factor to limit GP-1 production in a shake flask, which will be improved in a fermentor by optimizing agitation and aeration in the future.

## 4. Materials and Methods

### 4.1. Microorganism

*Streptomyces kanasenisi* ZX01 obtained from Research and Development Center of Biorational Pesticide, Yangling, China, was isolated from soil near Kanas Lake, Xinjiang Province, China. Strain ZX01 is registered at China General Microbiological Culture Collection Center (CGMCC) under strain number CGMCC 4893. The strain was maintained on Gauze’s No.1 agar plate and sub-cultured at a monthly interval, or stored in 20% glycerol at −70 °C.

### 4.2. Cultivation and Media

Seed inoculum was prepared by inoculating a loop of strain ZX01 growing on Gauze’s No.1 agar plate for 72 h into a 250 mL shake flask containing 100 mL seed medium. The flasks were incubated at 28 °C on a shaker at 150 rpm for 72 h.

An amount of 1% seed inoculum was inoculated into a 250 mL shake flask with 100 mL fermentation medium. All shake flasks were incubated at 30 °C and 200 rpm for 7 days.

The seed medium was (g/L): soluble starch, 20; KNO_3_, 1; K_2_HPO_4_, 0.5; MgSO_4_∙7H_2_O, 0.5; NaCl, 0.5; FeSO_4_∙7H_2_O, 0.01. The fermentation medium was (g/L): millet steep liquor, 10; soluble starch, 10; yeast extract, 3; NaCl, 2.5; CaCO_3_, 0.2.

### 4.3. Extraction and Determination of Glycoprotein GP-1 Production

The fermentation broth was centrifuged at 10,000 rpm for 20 min to separate the precipitate and supernatant. The supernatant was concentrated to a volume of 10 mL by a rotary evaporator and then precipitated by adding 4-fold volumes of ethanol at 4 °C. The precipitate was redissolved in distilled water (10 mL) and centrifuged (10,000 rpm, 10 min) again to remove those water-insoluble materials. The supernatant was subjected to DEAE-52 Cellulose anion-exchange column (2 cm × 60 cm) eluted with deionized water first, and then with 0.1 M NaCl at a flow rate of 5 mL/min. The 0.1 M NaCl fraction was collected and centrifuged (10,000 rpm, 15 min) with centrifugal filter devices (3 K, 0.5 mL) to remove NaCl. The fraction was subjected to HiTrap^TM^ Con A 4B eluted with binding buffer (20 mM Tris-HCl, 0.5 M NaCl, 1 mM MnCl_2_, 1 mM CaCl_2_, pH 7.4) and elution buffer (0.1 M methyl-α-d-glucoside, 20 mM Tris-HCl, 0.5 M NaCl, pH 7.4) sequentially at a flow rate of 1 mL/min. The fraction eluted with elution buffer that contained GP-1 was concentrated to 100 µL and analyzed by high performance liquid chromatography (HPLC).

The concentration of GP-1 was analyzed by a HPLC (Waters, Milford, MA, USA) with a gel filtration column (TSK-gel G2000SWXL, 7.8 × 300 mm, 5 µm, TOSOH, Tokyo, Japan) and a 996 photodiode array detector at 280 nm. HPLC was performed on a 10-µL sample with 20% acetonitrile at a flow rate of 0.5 mL/min and 28 °C. GP-1 purified previously (purity > 99%) was diluted to 10, 5, 2.5, 1.25 and 0.625 mg/mL as standards.

### 4.4. Determination of Dry Cell Weight

The precipitate obtained by centrifuging the broth sample was washed with distilled water twice, and then freeze-dried to a constant weight by a freeze dryer (SIM International Group Co., Ltd., New York, NY, USA), expressed as dry cell weight (DCW, g/L).

### 4.5. Determination of Anti-TMV Activity

The anti-TMV activity was tested using the half-leaf method. The fermentation broth diluted to one-twentieth was equally mixed with TMV (50 µg/mL). After 10 min, the mixture was mechanically inoculated onto the left side of the leaves of *Nicotiana glutinosa* as the treatment, while the right side of the leaves was inoculated with a mixture of distilled water and TMV as the negative control. *N. glutinosa* were kept in a culture chamber at 28 °C for 2–3 days, and then the number of local lesions on the leaves was recorded. The inhibition rate was calculated as follows:

(3) $$\mathrm{inhibition}\;\mathrm{rate}\left(\%\right)=\left(1-\frac{T}{C}\right)\times 100\%$$

where *T* is the average number of local lesions of treatment, *C* is the average number of local lesions of negative control. All experiments were conducted in triplicate. TMV was stored in systemic host *Nicotiana tabacum* K_326_ and purified as described by Gooding and Hebert [34].

### 4.6. Experiment Design by Response Surface Methodology

Response surface methodology based on central composite design was used to determine optimal levels of fermentation conditions. The concentrations of inoculum volume (*X*_1_), initial pH (*X*_2_) and rotating speed (*X*_3_) were selected as the independent variables (main factors). The levels of the variables are presented in Table 1. The total number of experimental combinations was estimated according to the equation:

(4) $$N=2^{k}+2k+n_{0}$$

where *N*, *k* and *n*_0_ are the number of experimental combinations, the number of variables and the number of repetitions of experiments at the central point, respectively.

A total of 20 experiments were performed according to Equation (2), including 2^3^ cube points, 6 axial points and 6 repetitions. The selected independent variables (*X*_i_) were coded as *x*_i_ according to the equation:

(5) $$x_{i}=\frac{X_{i}-{\bar{X}}_{i}}{\Delta X_{i}}\left(i=1,\;2,\;3,\ldots k\right)$$

where *x*_i_ is the coded value of the variable, *X*_i_ is the actual value of the variable, ${\bar{X}}_{i}$ is the actual value of the variable at the central point and $\Delta X_{i}$ is the step change value.

The mathematical relationship between the response variable (GP-1 production) and the independent variables can be described by the following equation:

(6) $$Y=b_{0}+\underset{i}{\sum }b_{i}x_{i}+\underset{ij}{\sum }b_{ij}x_{i}x_{j}+\underset{ii}{\sum }b_{ii}x_{i}^{2}$$

where *Y* is the predicted response, *b*_0_, *b_i_*, *b_ij_* and *b_ii_* are regression coefficients for the intercept, the coefficient of linear effects, the interaction coefficient and coefficients of quadratic effect, respectively. *x*_i_ and *x*_j_ are coded values of the independent variables (*i* < *j*).

### 4.7. Fermentation in Bench-Scale Fermentor

Bench-scale fermentation of *Streptomyces kanasenisi* ZX01 was performed in 5-L quadruple glass bioreactors (GBCN-5C, Zhenjiang East Biotech Equipment and Technology Co., Ltd., Zhenjiang, China) with a working volume of 3.5 L (Table 4). The bioreactors were equipped with a thermometer, pH sensor, dissolved oxygen sensor, tachometer, air-flow meter and internal pressure sensor and foam-sensing probe. The agitation system consisted of two impellers with four flat blades and a magnetic base. The agitation rate was controlled by electromagnetic impulse. The aeration system was an air inlet through a ring sparger with an air-flow meter and filter. Temperature was maintained constant by a heating system in the bottom and cooling water. The bioreactor and all its parts containing 3.5 L medium were sterilized by a high-pressure steam sterilization pot at 121 °C for 30 min. After sterilization, the fermentation medium was inoculated with seed inoculum. The signal of the foam-sensing probe was connected to an electromagnetic valve through a relay to add antifoam.

The optimal fermentation conditions obtained from the shake flask were applied in bench-scale fermentation, while the aeration rate and temperature were controlled at 1.0 vvm and 30 °C, respectively. Fermentation lasted for 7 days and samples were collected at every 24-h interval for measurement of GP-1 production and DCW. Dissolved oxygen (DO) and pH were monitored online and recorded over the fermentation process.

## Figures and Tables

**Table 1:** Levels of the variables by central composite design.

Factors	Variables	Levels of Variables				
		−1.682	−1	0	1	1.682
Inoculum volume (%)	*X*_1_	1.64	3.00	5.00	7.00	8.36
Initial pH	*X*_2_	5.3	6.0	7.0	8.0	8.7
Rotating speed (rpm)	*X*_3_	116	150	200	250	284

**Table 2:** Experimental design, experimental values and predicted values.

Run	*x*_1_	*x*_2_	*x*_3_	Experimental Values (mg/L)	Predicted Values (mg/L)	Error
1	1	1	−1	0.8276	0.802	0.0256
2	0	0	0	0.8315	0.830	0.0015
3	0	0	−1.682	0.8301	0.872	−0.0419
4	1	−1	1	0.6838	0.666	0.0178
5	−1	−1	1	0.6753	0.660	0.0153
6	0	0	0	0.8356	0.830	0.0056
7	0	0	0	0.8211	0.830	−0.0089
8	0	0	0	0.8151	0.830	−0.0149
9	0	1.682	0	0.5352	0.566	−0.0308
10	0	0	0	0.8332	0.830	0.0032
11	0	−1.682	0	0.7669	0.802	−0.0351
12	−1	−1	−1	0.8435	0.837	0.0065
13	1.682	0	0	0.7296	0.790	−0.0604
14	0	0	0	0.8490	0.830	0.0190
15	1	1	1	0.5567	0.517	0.0397
16	−1	1	−1	0.7317	0.704	0.0277
17	0	0	1.682	0.4760	0.486	−0.0100
18	1	−1	−1	0.9286	0.890	0.0386
19	−1.682	0	0	0.7027	0.703	−0.0003
20	−1	1	1	0.4558	0.474	−0.0182

**Table 3:** ANOVA of the model.

Source	Sum of Squares	Degree of Freedom	Mean Square	*F* Value	*P* Value
Model	0.34	9	0.0370	29.61	<0.0001
$x_{1}$	0.0083	1	0.0082	6.56	0.0283
$x_{2}$	0.0660	1	0.0660	52.43	<0.0001
$x_{3}$	0.1800	1	0.1800	140.81	<0.0001
$x_{1}x_{2}$	0.0013	1	0.0013	1.06	0.3278
$x_{1}x_{3}$	0.0006	1	0.0006	0.51	0.4917
$x_{2}x_{3}$	0.0022	1	0.0022	1.78	0.2119
$x_{1}^{2}$	0.0130	1	0.0130	10.47	0.0089
$x_{2}^{2}$	0.0410	1	0.0410	32.48	0.0002
$x_{3}^{2}$	0.0400	1	0.0400	31.63	0.0002
Residual	0.0130	10	0.0013		
Lack of fit	0.0120	5	0.0024	16.94	0.0037
Pure error	0.0007	5	0.0001		
Cor Total	0.3500	19			

**Table 4:** Specifications of the fermentor.

Fermentor	Bench-Scale
Total volume (L)	5
Working volume (L)	3.5
Diameter of fermentor (m)	0.15
Diameter of impeller (m)	0.07
Height of fermentor (m)	0.30
Baffle	3
Impeller	Two impellers with four flat blades
Type of drive	Magnetic stirred
Sterilization	Off-situ
